# Distance deviation measure of contouring variability

**DOI:** 10.2478/raon-2013-0005

**Published:** 2013-02-01

**Authors:** Peter Rogelj, Robert Hudej, Primoz Petric

**Affiliations:** 1 Faculty of Mathematics, Natural Sciences and Information Technologies, University of Primorska, Koper, Slovenia; 2 Institute of Oncology Ljubljana, Ljubljana Slovenia

**Keywords:** contouring, contour comparison, distance transform

## Abstract

**Background:**

Several methods that are currently used for contouring analysis have problems providing reliable and/or meaningful results. In this paper a solution to these problems is proposed in a form of a novel measure, which was developed based on requirements defined for contouring studies.

**Materials and methods:**

The proposed distance deviation measure can be understood as an extension of the closest point measures in such a way that it does not measure only distances between points on contours but rather analyse deviation of distances to both/all contours from each image point/voxel. The obtained result is information rich, reliable and provided in a form of an image, enabling detailed topographic analysis. In addition to image representation, results can be further processed into angular representation for compact topographic analysis or into overall scalar estimates for quick assessment of contour disagreement.

**Results:**

Distance deviation method is demonstrated on a multi observer contouring example with complex contour shapes, *i.e*., with pronounced extremes and void interior. The results are presented using the three proposed methods.

**Conclusions:**

The proposed method can detect and measure contour variation irrespective of contour complexity and number of contour segments, while the obtained results are easy to interpret. It can be used in various situations, regarding the presence of reference contour or multiple test contours.

## Introduction

Medical treatment often involves planning or analysis based on delineated structures defined on 3D images such as MRI or CT.^1^–^3^ One typical area is three dimensional image guided radiotherapy, which, on the basis of delineated structures, enables individualized irradiation, applying high doses to the target volume while respecting organs at risk dose constraints. The structures are defined by contouring regions of interest on 2D image slices, such that multiple contours on many slices may be required to delineate one structure of interest. The contouring requires highly skilled experts, because the boundary line of the structure may not be clearly visible or multiple edges may appear in the region. Furthermore, even small changes in contour position may have high impact on the success of the treatment and consistent reporting. Certain contouring analysis is therefore required to quantify inter and intra-observer variability, automatic contouring algorithms error or to compare contouring using different imaging technologies. The contouring error is difficult to assess, because the ground truth is generally not known. In some cases reference contours can be provided by an experienced (and eminent) expert or by consensus of a panel of experts. Otherwise, the magnitude of contouring variation between observers can be used as an indicator of contouring quality. The technical problem in evaluating contour variability is the absence of generally accepted method for contour comparison. Several methods were proposed, however certain limitations usually prevent their widespread use.

In this paper we focus on this problem and propose a novel measure of contouring differences, *i.e*., a distance deviation measure. Two different purposes of contour analysis need to be distinguished. The first one is analysis of contouring variation, *e.g*., for studying the contouring process. The metric for these studies must assess the difference between contours, which are drawn on image slices and are, thus, two-dimensional (2D) objects. Consequently, the analysis should also be performed in two dimensions only. On the other hand, if the purpose of the analysis is to study the effects of contouring variation, the whole delineation of a three-dimensional (3D) structure must be evaluated, which requires analysis based on three-dimensional metrics. Of course, both of the study types are interrelated; however, the relation between them may not be straightforward.

A review of methods for contouring analysis was made by Jameson *et al.*^4^ The most widely used method is comparison of structure volume. The major problem of this method is that even if two structures have the same volume, they may have different shape or location, which is not taken into account. The second method, with the opposite drawback, is comparison of the centre of volume (COV). It defines a single point that represents the whole structure and cannot distinguish structure size or shape. The next, very popular metric is a conformity index (CI), also called concordance or Jaccard index. It is defined as the percent ratio of the volume of intersection and volume of the union. To take into account more than two delineations, its generalized version was proposed (CIgen).^5^

The limitation of those measures is in their relativity. They do not provide any information of absolute variation in size, shape or location. All these metrics have an additional problem, that different implementation, *i.e*., using different volume computation methods, may yield different results.^4^ These measures are rarely used individually and are often combined to detect different kinds of contouring variations.^6^–^10^

The solution to the previously mentioned problems was often searched by methods that quantify shape or surface variations. The shape and surface differences were most often analyzed by mapping to cylindrical or spherical parametric space and observing the edges of structures from the origin.^11^ The problem here is in the definition of coordinate system origin and in exaggerated results when structures have complex non-circular or non-spherical shapes.

**FIGURE 1.**
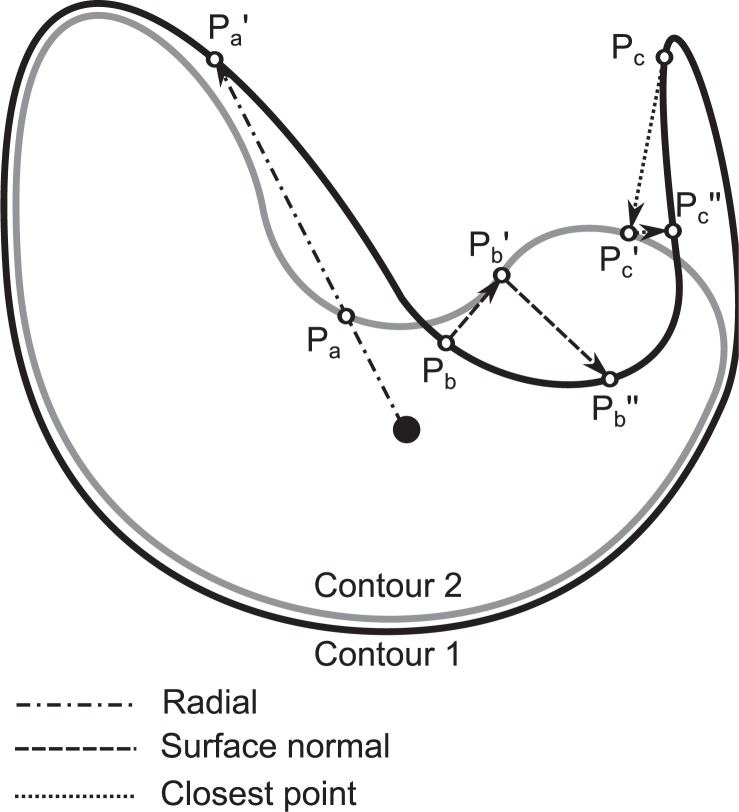
Illustration of limitations of methods based on origin, surface normal and closest points. The radial and surface normal methods may overestimate the distance between the contours. The surface normal and closest point methods are not symmetric, which means that distance may be different when measuring from contour 1 to contour 2 than it is in the opposite direction. Asymmetry also means that certain differences cannot be detected in certain direction, as some points on the contour may never get matched, e.g., point *P*c when measuring distance from contour 2 to contour 1.

Especially difficult is comparison of structures if the surface is not ’visible’ from the origin. There are two alternative methods, which measure distance between shapes or surfaces according to the surface normal^12^,^13^ or according to closest points.^14^,^15^ The problem here is the lack of symmetry; interchanging the first and the second structure yields different results. The most important drawback of asymmetric methods is that they cannot detect all the differences, especially in the case of complex shapes (Figure 1). The symmetrisation of the closest point method to obtain more reliable statistics could be made by applying it in both directions.^16^ However, due to differences between results obtained in both directions, spatial information about the contour correspondence is not obtained. These inconsistencies were reduced by ComGrad method^17^, which searches for the point pairs according to gradients of edges of both compared structures. However, the one-to-one mapping of all points on two different contours is in general not possible and limits the reliability of all these methods.

To design a new method for analysis of contour variations, the requirements of such measure need to be clearly defined. First, the measure must be expressed absolutely in standard distance units, *e.g*., millimetres, in order to enable meaningful interpretation of results, as the opposite to relative methods that provide some ratio that is less informative in the aspect of contouring differences. It needs to be consistent and therefore symmetric. Second, it must enable topographic analysis, *i.e*., measuring of local discrepancies irrespective of the complexity of contours. Third, it must be general enough to be usable for various kinds of contouring studies, *i.e*., for studies with or without the reference contour and for arbitrary number of test contours. The same principle must be appropriate for measuring in 2D or 3D space.

## Materials and methods

The proposed measure of contouring variations is based on the well-known closest point measure, however extended from measuring distances between contours, *i.e.*, distances between points on contours only, to measuring deviation of distances from each image point/voxels to all contours, *i.e*., for the whole image. This makes it symmetric and capable of meaningful measurement of arbitrary complex contour shape differences. The core of the measure is the Euclidean distance transform that has already proved its applicability in implementations of the closest point measure. The principle is explained in 2D space; however, the extension to 3D is straightforward as the only required difference is in the dimensionality of the distance transform used.

### Application of Euclidean distance transform

The well-known closest point measure compares two contours by measuring distances from points in analysed contour to closest points in the other, reference contour. Due to the asymmetry it was proposed to apply it in both directions.^18^ Such symmetrisation makes problems in localization of contour differences and does not fulfil our requirements. In spite of that, the closest point measure inspired the proposed measurement methodology by its implementation using the Euclidean distance transform.

The general idea was to measure distances between contours by computing Euclidean distance images.^19^,^20^ A distance image covers the same region as the original image on which the contours were drawn. It is created from contours defining the delineated structure such that the value of each image voxel equals the Euclidean distance from the voxel centre to the closest point on the contour. Its computation starts with drawing a delineation image **I**, which is a binary image with voxels inside the delineated structure represented by logical ’1’ and voxels outside the structure represented by logical ’0’. Then the distance image **D** is computed by application of the Euclidean distance transform. Here, each voxel is a real number representing a distance to the closest edge of the structure. Voxels outside the structures obtain positive values, while inside values are negative.

**FIGURE 2.**
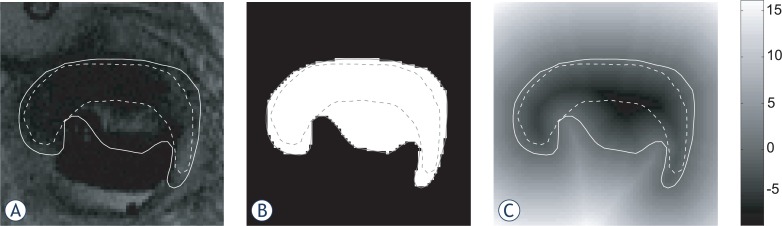
An illustration of computation and use of Euclidean distance transform for measuring closest point distances. A reference contour (solid line) and an evaluated contour (dashed line) were drawn on the original image (a). Using the reference contour a delineation image I (b) and distance image D (c) were computed. Closest point distances were obtained as an absolute value of the distance image for points in the evaluated contour. The colour scale of the distance image was provided on the right hand side and is given in millimetres.

When used for the closest point measure, the distance image **D** needs to be computed only for the first contour, *i.e*., the reference. The closest point distances are computed for points in the other, *i.e*., analysed contour. For a point x, the closest point distance equals the absolute value of the (reference) distance image in this point, |**D**(**x**)|. Due to the discrete nature of the distance image, some interpolation may be required. The accuracy of the obtained results equals the distance image voxel size. Generally, it is recommended to use the same voxel size as in the original image, because it also implies limitations of the contouring accuracy. Smaller voxels may be used if higher accuracy is required. See the illustration of computation and use of the distance transform (Figure 2).

When contours of 3D structures are compared, the distance transform is performed on each individual image slice separately, to preserve the 2D nature of contours. Alternatively, 3D distance transform could be used to compare 3D delineation volumes.

There are several techniques for effective computation of the Euclidean distance transform.^21^–^23^ However, their description or comparison is out of scope of this paper.

### Distance deviation measure

Our approach to avoid asymmetry and one-to-one mapping of contour points is to analyse distances to both/all contours from every image voxel. The difference between contours is therefore observed not only from the points on the contours, but also from the perspective of various nearby points. Using multiple points, covering the whole region of interest, any contour difference can be detected and measured not regarding the contour complexity. In addition, such approach enables localization of contour differences in a sense of their influence on the nearby regions. The difference of two contours **C**_1_ and **C**_2_, where one of the contours is considered as a reference for evaluating the other contour, and from the perspective of some point with coordinate **x** can be measured using the corresponding Euclidean distance images **D**_1_ and **D**_2_ as their absolute difference:

[1] $$\begin{array}{lll} DD_{12}(x) & = & \sqrt{{\left(D_{1}(x)-D_{2}(x)\right)}^{2}}\\ & = & abs\left(D_{1}(x)-D_{2}(x)\right) \end{array}$$

Let us call the result *DD*_12_(**x**) a distance deviation. It denotes a deviation of distances from an observation point to both contours. Note that distance images **D** of both contours are needed in contrast to the closest point **x** measure where distance image is computed only for one of the contours. The measure is obviously symmetric, such that *DD*_12_(**x**) = *DD*_21_(**x**) = *DD*_R_(**x**).

Furthermore, each difference between contours can be detected by selecting an appropriate observation point **x**. Therefore, by analysing the distance deviations from all surrounding points, no difference between the contours remains undetected. The results denote contour differences in the absolute manner, e.g., in millimetres, and enable meaningful topographic analysis.

Contouring studies often require analysis of multiple contours, *e.g*. to find some general characteristics of contour disagreement. A reference contour may be provided to define the ground truth. In such cases distance deviation measure estimates the contour disagreement for observation point x as a RMS value of distance deviations corresponding to individual analysed contours with respect to the reference one:

[2] $$DD_{R}(x)=\sqrt{\sum_{i=1}^{N}\frac{{\left(D_{i}(x)-D_{R}(x)\right)}^{2}}{N}}$$

Here, **D**_R_ is a reference distance image and N is the number of analysed contours. Note that distance image **D**_i_ must be computed for every contour C_i_ involved into analysis.

When the reference is not known, the contour error can not be measured. However, the magnitude of contouring variation can be used as an indicator of contour quality. The contouring variation can be estimated by computing distance deviations against an average contour instead of the reference one. The average contour does not need to be computed, because only the average contour distance image is needed and can be obtained by averaging distance images of all analysed contours:

[3] $$\bar{D}(x)=\frac{\sum_{i=1}^{N}D_{i}\left(x\right)}{N}$$

Distance deviations, in the case of absence of reference labelled *DD_S_*, are then obtained using exactly the same principle as in the case of provided reference, and for some observation point **x** represent a standard deviation of distances form this point to all analysed contours **C**_i_:

[4] $$DD_{S}\left(x\right)=\sqrt{\sum_{i=1}^{N}\frac{{\left(D_{i}\left(x\right)-\bar{D}\left(x\right)\right)}^{2}}{N}}$$

To summarize, every contour discrepancy can be detected by measuring deviation of distances from nearby points to all analysed contours, and when deviations for all image points are computed, no contour deviation can remain undetected, even in the case of complex and pronounced extremes or void interior. It is also important that there is no need to perform any mapping of points on different contours and still obtain detailed topographic information of the contour differences. The proposed distance deviation measure can be used in various situations, not regarding the existence of the reference contours, nor the number of analysed delineations. We recommend computing distance deviations in all image points, *i.e*., all image voxels, because the distances to contours can be obtained by the distance transform and the points are dense enough to reliably detect all contour differences. The accuracy is still limited by the accuracy of distance images and can be improved using higher resolution distance images, such as in the case of the closest point measure. Interpolation is not required, because points defined by image voxels are used instead of contour points that are used in the closest point measure.

### Presentation of results

A clear representation of results that is understandable to the observer has important implications. Among other, it may have a significant positive effect on the learning process. In this way, it enables the observer to improve his contouring, which can be expected to result in improved treatment results and consistent and comparable treatment recording and reporting.

In order to interpret the obtained results, they also need to be presented in a form familiar to the observer and compliant with requirements of each particular study. We have identified three different representation methods that could satisfy all common study requirements:

- image representation,
- angular representation, and
- overall scalar estimates.

The later two methods require some statistical analysis to depict information rich results in a more compact form. Because result representation methods can be used irrespective of the presence of reference contour, we use *DD* as a general notation for distance deviations, computed either as *DD*_R_ [2] or as *DD*_S_ [4].

### Image representation

The most informative way of presenting differences between contours is to visualize them on the original medical image that was used to draw the contours. The distance deviation measure provides the information of contouring variability in a form of an image and can be easily visualized in such a way. The advantage of visualizing distance deviations as an image with respect to visualizing the contours only is in explicit labeling of impact of contouring differences to nearby regions. This information enables observer not only to see the different contours but also to easily judge them from the perspective of their influence to nearby structures.

Medical images are typically provided as a grid of scalar measurements that are visualized as pixel intensities, *i.e*., gray values. The colour spectrum is not used, except for visualizing additional data, *e.g*., contours or labels. Thus, the extension of gray values to a colour space can be used to jointly visualize both, information of the original medical image as well as the contour variability.

There are several ways to colour code the contour disagreement as well as preserve the displayed information of the original medical image. One of them is to use a HSV (hue, saturation, and value) colour space.^24^ To preserve the well known representation of medical image by intensity values, this information shall be coded as the value component (V). The contour disagreement is most clearly understandable when coded as the hue component (H), *e.g*., representing low contour disagreement with blue and high disagreement with red color. The saturation component is not used to present any information and shall be high enough to well distinguish between the other two components.

**FIGURE 3.**
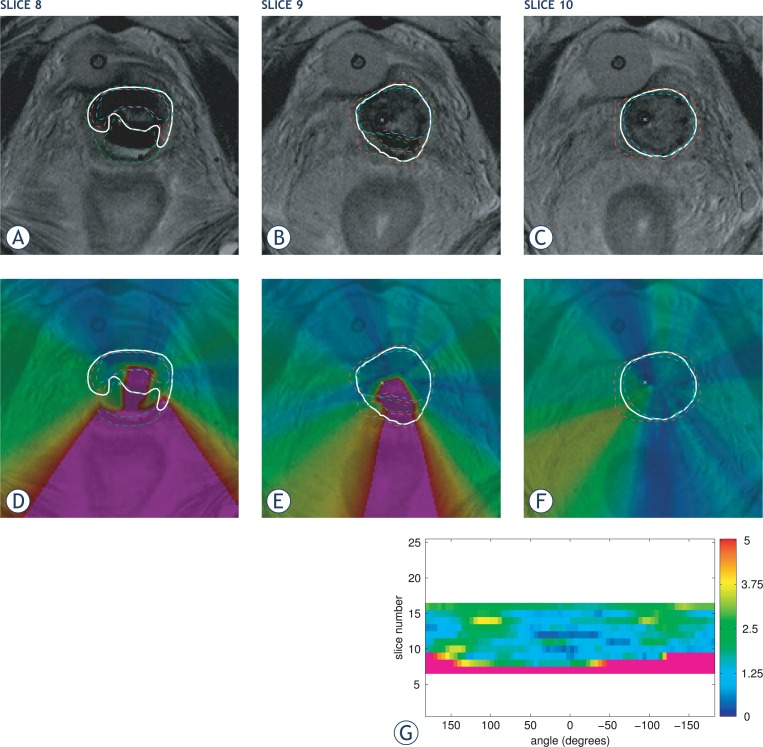
An example of contouring analysis from the field of brachytherapy. The reference contours are in images shown with thicker white line and the analyzed contours with thinner dashed ones. The small cross represents the center of the uterine tandem and is used as the contour center for the angular analysis. The top row (a–c) shows selected slices of the original image, the second row (d–f) is image representation of distance deviations and at the bottom (g) is an angular representation of distance deviations for all image slices. Here, the angle of zero degrees corresponds to the anterior direction (top of the image), negative angles to the right hand side of the body (right hand side of the image) and positive values to the left hand side of the body (left hand side of the image). The color scale of image representation equals the one of angular representation; black/purple represents contour discrepancy greater than 5mm. The overall maximal distance deviation (*DD_R,max_*) equals 17.96 mm and the mean distance deviation ( ${\bar{DD}}_{R}$) equals 2.17 mm.

An example of image representation of contour disagreement measured by distance deviations *DD*_R_ is shown in Figures 3d–f.

### Angular representation

In order to present distance deviations in a more compact form that enables topographic analysis of a whole 3D structure from a single graph, they can be shown using angular representation in cylindrical coordinate system defined by some contour center. Angular representation is often used for topographic analysis of contouring variations.^25^–^27^ In the case of distance deviations, the contour differences are presented by maximal distance deviation observed on certain image slice and in certain direction from the contour center. For example, an angle of 0 degrees typically represents the anterior part of the structure, +90 degrees left, −90 degrees right and ±180 degrees correspond to the posterior direction. The contour center may be defined as a center of gravity or according to position of other geometrical features, *e.g*., applicators or needles.

Such representation is convenient for circular structures, while for more complex shapes it may blur the spatial information. However, although the spatial information may be blurred, the presented results are still correct and are not exaggerated like they are when using circular or spherical coordinate space directly for the analysis.

Converting an image of distance deviations into angular form requires some post processing. For each angle, the maximal distance deviation is searched from the contour center to the edge of the region of interest. However, it turns out that distance deviations in regions outside the most distant contour can not exceed the ones inside that region. Similarly, distance deviations inside the innermost contour are also reflections of some contour disagreement that can be detected in area between the innermost and the outermost contour, for a proof see the appendix. Thus, we can limit our analysis only to this region of contour disagreement Δ*I*:

[5] $$\Delta I\left(x\right)=\{\begin{array}{cc} 1 & \text{if}\;0<\sum_{i=1}^{M}I_{i}\left(x\right)<M\\ 0 & \text{otherwise} \end{array}$$

Here, M is a total number of contours including the reference one, if available. Limiting the analysis to this region also avoids misinterpretation of angular results, because distance deviation in some point in the inner region does not necessarily represent disagreement of contours in the direction of this point from the contour center. This is especially evident in the case of eccentric or complex contour shapes. For the example see Figure 3, slice 9.

The maximal angular distance deviation in the observed region Δ*I*, can be obtained using cylindrical coordinate system, where r and ø denote the radial distance and the angle according to the contour center:

[6] $$DD∠\left(\phi \right)=\underset{\phi \in \left(-\pi ,\pi \right),\;r\in \left(0,\infty \right)}{\text{max}}\;\left(DD\left(r,\phi \right)\cdot \Delta I\left(r,\phi \right)\right)$$

Depending on the angle discretization, some interpolation of distance deviation image *DD* may be required.

Angular representation is more compact than image representation, providing only the maximal contour disagreement in certain part of the analyzed structure without detailed distribution of contour disagreement with respect to patient anatomy. However, due to compactness, results of multiple slices can be presented in a single graph to describe the contouring variations for a whole surface of the 3D structure. An example of angular representation is shown in Figure 3G.

### Overall scalar estimates

For a quick estimate of contour disagreement a single scalar value representing the overall score is often required, although such representation does not enable topographic analysis.

Different statistical methods were proposed to compact complex and information rich results into a single representative value. In general, maximal and mean values are commonly used. Maximal value of a distance metrics is also known as a Hausdorff distance^28^ and is popular for evaluating segmentation methods.^13^,^16^,^29^ Maximal distance deviation may be obtained by searching over the whole 3D distance deviation image. The same result can be obtained by searching for maximum only in the regions of contour disagreement Δ*I*:

[7] $$DD_{\max}=\underset{\Delta I\left(x\right)=1}{\text{max}}\left(DD\left(x\right)\right)$$

Here, the result is a maximal contour discrepancy, which equals the Hausdorff distance for M = 2. In this specific case the value in the interior of the contours cannot exceed the maximum on the contours. Alternatively, the contour disagreement could be represented by a mean value of distance deviations in some region. It can be obtained by averaging in different domains, e.g. according to area in the image representation or according to angle in the angular representation. Each approach lays stress on different properties of contour disagreement and has limitations on the others. Each one of them may be ambiguous in some perspective and may, due to information reduction, not clearly depict contour differences of complex shapes.

We have found averaging according to the area in the delineated regions to be the most balanced one. Here, the region for averaging *Î* is defined by union of all delineated regions *I*_i_ corresponding to individual contours including the reference one:

[8] $$\hat{I}\left(x\right)=\{\begin{array}{cc} 1 & \text{if}\;\sum_{i=1}^{M}I_{i}\left(x\right)>0\\ 0 & \text{otherwise} \end{array}$$

The union region enables balanced quantification of contour variability with respect to the whole delineated structure, without excluding eventual high contouring errors at outermost and innermost contours and parts of good contour agreement that reflects in low distance deviations in the interior of the region of contouring disagreement Δ*I*. Here, all the image slices must be considered in order to evaluate contours representing three dimensional structures. The overall estimate of contour disagreement in a form of an average distance deviation is

[9] $$\bar{DD}=\frac{\sum_{x}DD\left(x\right)\hat{I}\left(x\right)}{\sum_{x}\hat{I}\left(x\right)}$$

Note that average distance deviation in contrast to other distance deviation indexes may violate the triangular inequality requirement of a mathematical metric, and thus cannot be used to compare contours indirectly. The obtained maximal and average distance deviations are extremely compact. They provide absolute results, and enable quick insight into contouring variation for multiple contours. They provide different information and may in some cases yield opposing results.^30^ This makes them supplementary to each other.

## Results

To illustrate the distance deviation measure it was applied to a manually selected complex contouring example from the field of cervix cancer brachytherapy. The contouring was performed on MR image with voxel size 0.625 × 0.625 × 3.900 mm. The contours of three observers were analysed with respect to reference delineation. Contours were provided for all relevant image slices, *i.e*., for slices 7 to 16. The contours corresponding to three successive image slices 8, 9 and 10 are shown in a top row of Figure 3.

The complexity of the case is high due to topology of contours that include noncircular shapes with pronounced extremes (slices 8 and 9) and void interior (slice 8). The results are presented in two graphical forms; image representation and angular representation. Furthermore, the two proposed overall scalar estimates of contour disagreement are computed; maximal and average distance deviation.

For the image representation of contour disagreement using distance deviation measure see Figures 3D–F. The colour coding follows the colour scheme used in Figure 3G. The maximal distance deviation displayed is limited to five millimeters, larger values are coded with black/purple. Distance deviations are computed for each image pixel/voxel and thus enable detailed topographic analysis, including localization of (anatomical) regions that could be highly affected by contour differences.

In the provided example a large distance deviation can be noticed in central and posterior regions of slices 8 and 9. Results for these two slices also show that the presence of void interior regions and pronounced extremes does not limit capabilities of the measure to clearly and correctly evaluate contour differences.

The angular representation of distance deviations is presented in Figure 3G. Here, the results of each slice contribute one row in the graph, which, as such, provides the results for the whole image. For slices on which contours were not drawn the graph remains empty/white.

The colour scale represents distance deviations from zero to 5mm, larger values are coded with black/purple. Focusing on the selected slices, large distance deviations can be noticed for slices 8 and 9. However, the results provided in the angular form are not that unambiguous as in the form of an image, and slice 8 is a good example in that manner. Because the axis for angular analysis is in the region of contour disagreement and not in the interior of all contours, the high distance deviation around the contour center results in high values for multiple angles, in our case angles below −40 degrees. These values are normally related to the contour parts at the left hand side of the analyzed image region, where in our case contours match considerably well. The high values therefore indicate an error not only in the given part of the contours, but also in regions close to the contour center that are not in the interior of all contours. The position of the contour center has in such complex cases high influence on the angular results, which may make its positioning difficult if not defined anatomically. When contour shapes are less complex, the angular representation is unambiguous, such as in slice 10 of our example.

The overall scalar estimates of contour disagreement are not only more compact, but also less informative. The average distance deviation ( $\bar{DD}$), in our case ${\bar{DD}}_{R}=2.17\;\text{mm}$, is not optimal for detecting high local variations. A relatively low value means that contours are in good mutual agreement, but not also that there are no high local differences. On the other hand, maximal distance deviation (*DD*_max_), in our case *DD*_R,max_ = 17.96 mm, does not give a good insight into the overall contour agreement, but gives a clear warning when large local discrepancies are present. Thus, our example demonstrates the importance of both scalar estimates and their complementarity.

## Discussion and conclusions

In comparison to other measures, see the introductory section, the distance deviation measure satisfies all the requirements of contouring variability studies. The contour differences are measured absolutely with results given in millimeters enabling straightforward interpretation. The symmetry is assured by equal treatment of all analyzed contours and the reliability by observing contour differences from the perspective of contour surrounding, *i.e*., all image points/voxels, such that any kind of contour differences can be detected and measured.

The proposed novel measure of contour discrepancy was developed as a solution to problems identified in other measures. It extends the measurement of distance between two contours to the measurement of deviation of distances to both/all contours from points in nearby regions. The basic idea to follow the relation between contours and the image has a physical background; contouring errors influence the dose delivered to imaged regions, *i.e*., to the target as well as organs at risk. In radiotherapy, for example, contouring uncertainties result in uncertainties of dose distribution in nearby tissues and, therefore, have clinical consequences. Distance deviation measure does not tend to model the real influence of contouring errors to treatment of nearby regions. Instead, it uses this principle to gain robustness and reliability of detecting and measuring contour differences irrespective of contour complexity. Furthermore, this principle enables results to be easy to interpret, also because all contour differences are measured in standard distance units (millimeters). The impact of the image and angular representation of the index proposed here is in its capacity to allow the observer to appreciate the delineation uncertainties on anatomical images in the actual contouring plane, instead of a virtual 3D volume.

The distance deviation measure is suitable for diverse measurement tasks, not regarding the number of compared contours or presence or absence of a reference contour. The core of the measure is the Euclidean distance transform, which converts each analyzed contour into a distance image. Distance deviations are then obtained by basic statistical analysis (e.g. computing standard deviation) of obtained distances for each image voxel. The results can be presented clearly using different presentation methods tailored to the common needs of different contouring studies; from detailed topographic analysis to the compact scalar representation of the overall contour discrepancy.

Planning target volume (PTV) is a geometrical concept, derived from the clinical target volume (CTV) by applying margins around it to compensate for the effects of organ/patient movement, and uncertainties in beam and patient setup. The CTV as an anatomical/biological concept needs to be selected and delineated by the treating physician, before selecting the treatment modality and technique. According to these definitions, generation of margins from CTV to PTV to account for delineation uncertainties may not be advocated. Instead, every effort should be made to reduce these uncertainties through the use of adequate imaging, delineation guidelines and training.

The method has already proved its applicability in different studies of contouring in radiotherapy.^31^–^33^ Although we found no limitations in the measure itself, some precaution is needed when compact result representations are used. Compacting of results reduces the amount of information they provide and, thus, some information gets lost. Angular representation discards the information of the radial location component and makes the results sensitive to selection of the axis used for the analysis. Scalar estimates discard all the spatial information by averaging or by searching the maximum value in the region of interest. Nevertheless, the information provided by results of distance deviation measure, even in the case of the most compact scalar representations, enable reliable assessment of the overall contouring variability. As such, we believe in contribution of distance deviation measure to advancement of contouring studies.

## Figures and Tables

**FIGURE 4.**
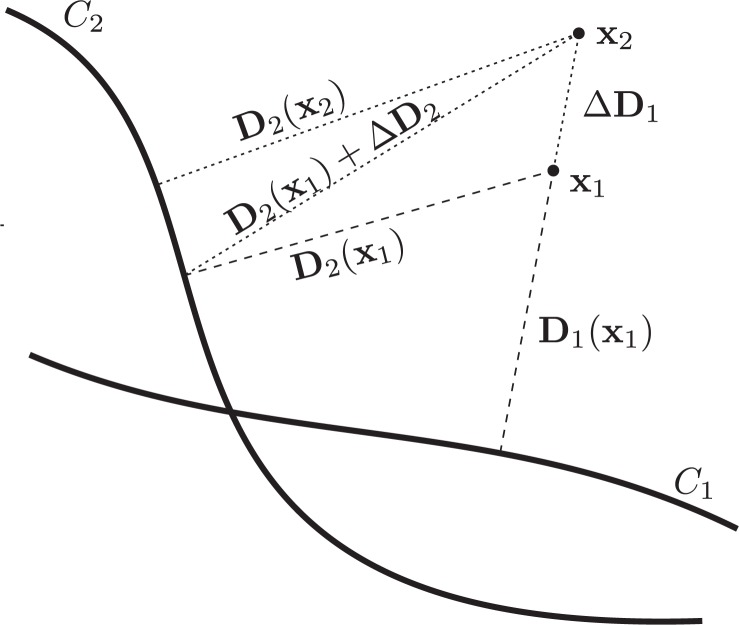
Illustration of distances for points outside the outermost contour.

## Appendix: Distance deviations outside the outermost contour

Let us assume that we have two contours C_1_ and C_2_ and some point x_1_ outside the contours such that distance **D**_1_(**x**_1_) is smaller than **D**_2_(**x**_1_), i.e., the the point **x**_1_ is closer to contour C_1_ than C_2_. For the illustration see Figure 4. Furthermore, a point **x**_2_ is positioned further from the contours on a line through **x**_1_ and its closest point on C_1_, such that **D**_1_(**x**_2_) > **D**_1_(**x**_1_). Let us compare distance deviations of these two points, i.e., *DD*(**x**_1_) and *DD*(**x**_2_). When moving form point **x**_1_ to point **x**_2_, the distance to the closest point on contour *C*_2_ cannot increase more than distance to contour *C**_1_*, due to triangular inequality:

[10] $$D_{2}\left({\text{x}}_{1}\right)+\Delta D_{2}\leq D2\left({\text{x}}_{1}\right)+\Delta D_{1}$$

As the distance deviation measure relies on the closest point distances, we have to be aware of the possibility that there may exsist some other point on *C*_2_ that is even closer to x_2_ than the original closest point of x1:

[11] $$D_{2}\left(x_{2}\right)\leq D_{2}\left(x_{1}\right)+\Delta D_{2}$$

and according to [10]

[12] $$D_{2}\left(x_{2}\right)\leq D_{2}\left(x_{1}\right)+\Delta D_{1}$$

Using the Eq. [1] and the observed relationship, we get:

[13] $$DD_{R}\left(x_{2}\right)=\text{abs}\;\left(D_{1}\left(x_{2}\right)-D_{2}\left(x_{2}\right)\right)$$

[14] $$=D_{2}\left(x_{2}\right)-D_{1}\left(x_{2}\right)$$

[15] $$\leq \left(D_{2}\left(x_{1}\right)+\Delta D_{1}\right)-\left(D_{1}\left(x_{1}\right)+\Delta D_{1}\right)$$

[16] $$=DD_{R}\left(x_{1}\right).$$

The obtained relationship *DD*_R_(**x**_2_) ≤ *DD*_R_ (**x**_1_) proves that distance deviation outside the outermost contour can not exceed distance deviation inside contours. This relationship is valid irrespective of the number of contours and holds also for the DD_S_ measure [4]. Similar relationship could be shown for the regions inside the innermost contour, such that it can be concluded that distance deviations in these regions are reflections of distance deviations in the region of contour disagreement Δ*I*(**x**).
